# Lipid Drug Carriers for Cancer Therapeutics: An Insight into Lymphatic Targeting, P-gp, CYP3A4 Modulation and Bioavailability Enhancement

**DOI:** 10.34172/apb.2020.064

**Published:** 2020-08-09

**Authors:** Shashank Chaturvedi, Anurag Verma, Vikas Anand Saharan

**Affiliations:** ^1^Department of Pharmaceutics, Institute of Pharmaceutical Research, GLA University, Mathura, Uttar Pradesh, India.; ^2^Department of Pharmaceutics, School of Pharmaceutical Sciences, IFTM University, Moradabad, Uttar Pradesh, India.; ^3^Department of Pharmaceutics, School of Pharmaceutical Sciences and Technology, Sardar Bhagwan Singh University, Dehradun, Uttarakhand, India.

**Keywords:** Lipid based carriers, Lymphatic system, Metastasis, P-gp, CYP3A4

## Abstract

In the treatment of cancer, chemotherapy plays an important role though the efficacy of anti-cancer drug administered orally is limited, due to their poor solubility in physiological medium, inability to cross biological membrane, high Para-glycoprotein (P-gp) mediated drug efflux, and pre-systemic metabolism. These all factors cumulatively reduce drug exposure at the target site leading to multidrug resistance (MDR). Lipid based carriers systems has been explored to overcome solubility and permeability related issues of anti-cancer drugs. The lipid based formulations have also been reported to circumvent the effect of P-gp and CYP3A4. Further long chain triglycerides (LCT) has shown their ability to access Lymphatic route over Medium Chain Triglycerides, as the former has been extensively used for targeting anti-cancer drugs at proliferating cells through lymphatic route. Therefore this review tries to reflect the usefulness of lipid based drug carriers systems (viz. liposome, solid lipid nanoparticle, nano-lipid carriers, self-emulsifying, lipidic pro-drugs) in targeting lymphatic system and overcoming issues related to solubility and permeability of anti-cancer drugs. Moreover, we have also tried to reflect how critically lipid based carriers are important in maximizing therapeutic safety and efficacy of anti-cancer drugs.

## Introduction

Cancer can be regarded as a multiplex of disease states generated with an outcome of prolonged injuries at tissue and cellular level through interactions with cancer causing agents known as the carcinogens. The administration of tobacco (people consuming tobacco products, or are in the vicinity of tobacco smoke), irradiation with ultraviolet light (ultraviolet-A produces genetic damage and suppression of immune system whereas ultraviolet-B has penetrability to the epidermis an evident factor for skin melanoma) and various viral infections which may integrate by incorporating their genetic material into the host’s DNA leading to mutation (hepatitis B virus- liver carcinoma, human papillomavirus- causative agent for cervical carcinoma, human herpesvirus 8- significantly affecting the integumentary system), epigenetic changes (alteration in gene expression without any significant change in DNA sequence namely DNA-methylation and histone modifications) and global transcriptome changes (through inflammatory pathways) these all factors increases the probability of converting healthy cells into cancerous cells.^1^ Cancer cells proliferate in the human body by gaining access into the blood vasculature and lymphatic streams, producing secondary tumors through metastasis.Strategically the treatment of patient suffering from cancer is undertaken through removal of cancerous or suspected tissues employing surgical intervention which is normally a first line approach accompanied by targeted radiation therapy to kill cancerous cell and reduce the size of the tumor, and finally systemically delivering the anti-cancer and immunomodulatory agents by chemotherapy and immunotherapy. A conventional chemotherapy strategy delivers anti-cancer drugs not only to cancer cells but also have access to normal healthy cells thereby producing plethora of side effects. Therefore, several attempts have been made in this regard to target anti-cancer agents at tumor sites by researchers in the last forty years through newer delivery approaches, among them lipid based drug delivery systems have gained sufficient interest, as these systems can address the complexity of anti-cancer drug delivery safely and effectively to localized tumors as well as in the vicinity of metastatic sites.^2^ This review is an attempt to address the role of lipid based drug carriers in lymphatic route drug targeting (an alternative voyage for delivering anti-cancer agents to metastatic cancer cells), avoiding hepatic first pass metabolism (cytochrome P-450), circumventing the effect of p-glycoprotein pump (as drug effluxer), we have also compared other nanoparticulate delivery system (polymeric and metallic) with lipid based nano-carriers in terms of formulation characteristics and their clinical interpretations.

## Challenges and opportunities in targeting anticancer drugs

On a cumulative note while addressing the severities of cancer, tumor metastasis accounts for largest percentage of adversities and mortalities. Cancer cells gains access to lymphatics by invading the broad intercellular pores just adjacent to endothelial cells together with irregular basement membrane.^3,4^ The cancer cell after gaining access to lymphatics uses it as a reservoir site, which results in further advancement of carcinoma distant to primary site of malignancy.^5-7^

## Role of lymphatic route

The lymphatic system is a nexus that comprises of lymph nodes, lymphatic vessels, spleen, thymus, Peyer’s patches and tonsils which function in coordination with the circulatory system and a circulating fluid called lymph. Lymph serves the purpose of transporting lymphocytes and antigen presenting cells into the blood and bones via lymph nodes. Further, the lymphatic system serves the purpose of maintaining the homeostasis by regulating the balance of water in body through transporting cells of the immune system back to the nodes of lymph and making delivery of extracellular fluid again in the central circulation.^8,9^ The potential aspects of Lymphatic drug transport for immunomodulatory agents and anti-cancer drugs using lipid based drug carrier systems have been explored by the researchers, as the lymphatic system serves as a pathway for lymphocytes particularly T and B and tumor metastasis.^10-17^ As the structure of lymphatic capillaries is extensively porous owing to their single layer of non-fenestrated endothelial cells which in turn allows the access of large particles.^18^

**Figure 1:**
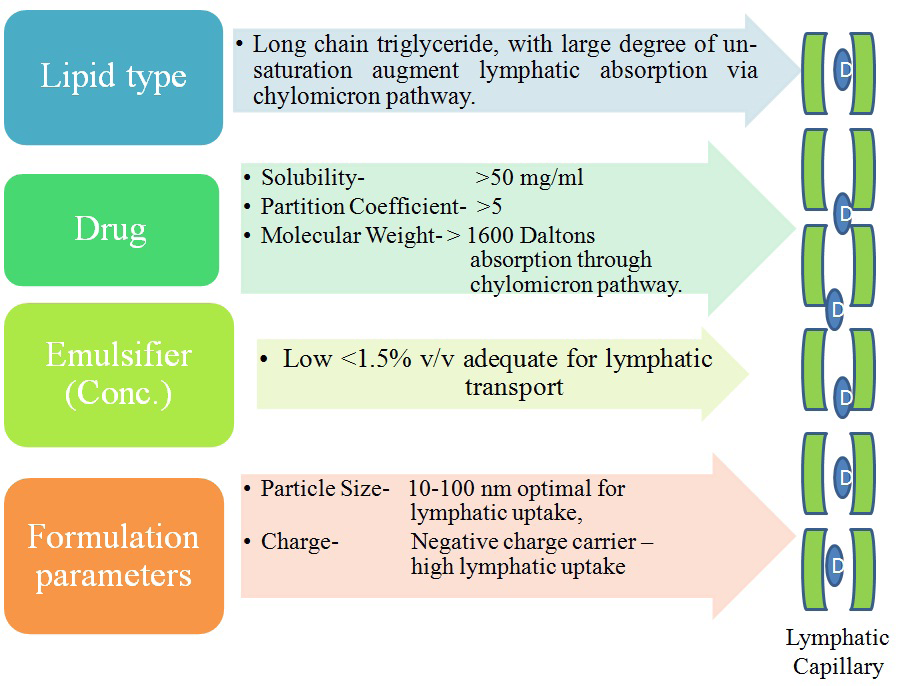
Schematic representation of key factors for lymphatic drug targeting by lipid based delivery systems (D= Drug reaching to lymphatic circulation).

Among the other factors like lipid composition, effective potential on the oil globules, molecular weight, dose, and particle size, the drug *in-vivo* behavior is more closely related with the particle size. As nano size particles persist larger surface area as compared to macro sized, therefore overall interaction with the biological membranes is enhanced providing better absorption into the lymphatic system, therefore it has been advocated that particles below 200 nm are effectively targeted into the lymphatic route. Charge on the formulations also governs the passage through luminal membrane having negative potential as a whole owing to glycosaminoglycans presence, therefore it is advocated that formulation those are neutral or bearing negative charge can be effectively transported into the lymphatic system as they can easily avoid electrostatic interaction compared with formulations having positive charge that persist stronger affinity towards the luminal membrane hence get retained.^19^ Key factors responsible for effective lymphatic route targeting are represented in Figure 1.

## Lymphatic transport of lipid products

The membrane-bound transporter proteins play a pivotal role for the transport of lipid digested products either by active or passive transport. The lipid transportation is advocated by enterocyte’s apical membrane post-parandially, as this is the time when lipid concentration in the lumen is substantially high which in turn promote passive transportation.^20-22^ The mechanistic approach at microvillus for fatty acid uptake may be related with sodium concentration directly or indirectly together with inclusion of fatty acid binding proteins and transporters.^23-25^ The intracellular transformation of lipid subsequent to enterocyte’s absorption is governed by its chain length. Lipids with short and medium carbon chain length (i.e. having C<12) generally spread beyond the enterocytes into the capillaries for gaining access to portal vein, whereas lipid having long-chain (i.e. C≥12) gain access to mesenteric lymph after migrating from the site of absorption with subsequent re-esterification and fabrication by the endoplasmic reticulum into the lipoproteins of the intestine (chylomicron and very low density lipoproteins), here chylomicron has direct relationship with lipid load.^26-29^ The magnitude of lipid access to lymphatic transport is not merely related to lipid chain length but also to its preference towards lymph (i.e. log P>5).^30,31^ Vahouny & Treadwell^32^ concluded from their experiments that co-administration of long chain triglycerides (LCT) increased lymphatic transport of cholesterol.Nankervis et al^33^ demonstrated the effectiveness of linoleic acid and cottonseed oil (Long chain lipids) for enhancing the lymphatic absorption of retinoids over Miglyol 812, (medium-chain lipid) the latter though were less sensitive to oxidation and have comparatively higher solvent capacity.^34-38^

Oral administration has always been attempted largely as compared with other routes considering potential benefits of patient’s compliance and cost effectiveness. But oral anti-cancer drug access to systemic circulation and at the target site suffers from multitude of factors like poor aqueous solubility, extensive pre-systemic metabolism, efflux transporter activity, and these all factors collectively accounts for resistance against anti-cancer drugs.

## Pre-systemic cytochrome based drug metabolism and P-glycoprotein mediated drug efflux

**Figure 2:**
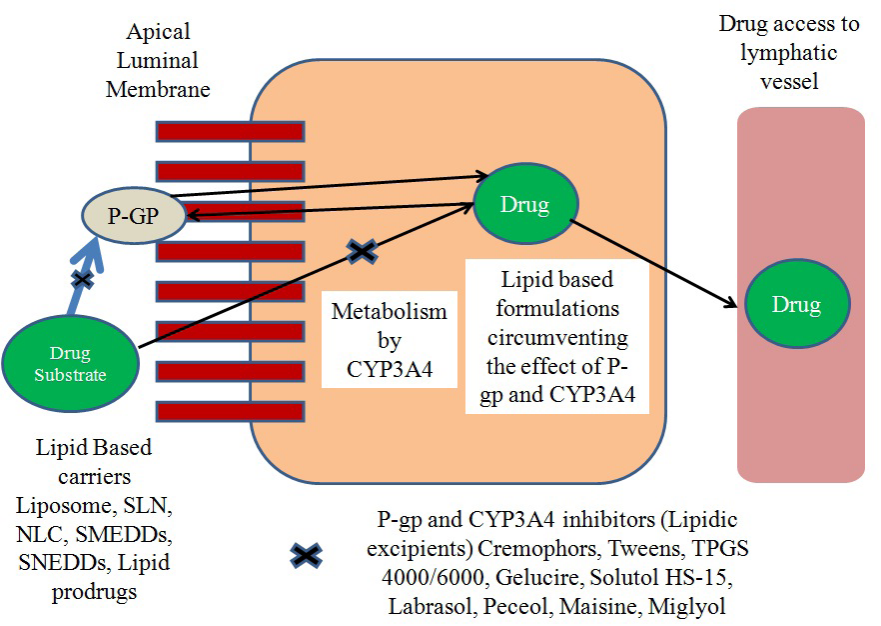
Schematic representation of role of lipid excipients in circumventing the effect of P-glycoprotein pump and Cytochrome 3A4 mediated drug efflux and metabolism.

P-glycoprotein pump (multi-drug effluxer) and Cytochrome P450 (drug metabolizer) perhaps work concomitantly as a protective barrier and are the most evident factors for reduced bioavailability of BCS Class II and IV drugs.^39-42^ The first pass effect on drug metabolism is a summation of enzymatic metabolism at brush border (alkaline phosphatase, isomaltase and sucrase and peptidases) of the lumen and at the intracellular level by microsomal enzymes on the endoplasmic reticulum in the cytoplasm.^43^ Cytochrome P450 particularly CYP 3A4, are the major contributors in phase I metabolism and accounts for oxidation of many anti-cancer agents, thereby reducing the bioavailability of drugs with ester moiety like capecitabine.^44,45^ The fraction of drug absorbed from the gastrointestinal tract reaches to liver (metabolizing house) through entero-hepatic circulation and undergoes biotransformation. This is known as first pass hepatic metabolism and it is the major contributor for lower oral bioavailability of many anti-cancer agents like Tamoxifen substrate for CYP3A4.^46,47^ The tandem situation becomes more critical when a drug is a substrate for both CYP3A4 and p-glycoproteins (p-gp).^48^ After the discovery of P-gp transporters (from the family of ATP-binding cassette transporters) expressed at carcinogenic cells, blood-brain barrier as major obstructing and excretory tissues by Juliano and Ling in the late 1970s, further studies started in establishing the role of P-gp as anti-cancer effluxer. Anti-cancer agents which are substrate to P-gp have compromised effectiveness owing to their altered pharmacokinetics. This condition is a major contributor in multidrug resistance (MDR). Furthermore, as the drug is effluxed out by p-gp, and is now again in the vicinity of CYP3A4 to show its metabolizing affect, the drug’s bioavailability gets synergistic reduction.^49-54^ A number of strategies to tackle the poor bioavailability due to synergistic or lone effect for drug which is substrate to either CYP3A4 or P-gp or both have been investigated. The concomitant use of P-gp inhibitors, surfactants and polymer with anti-cancer agents in the formulations are some of the most commonly explored approaches. Sandhu et al^55^ prepared SNEDDs of paclitaxel (PTX) with co-administration of curcumin. Sesame oil having high polyunsaturated fatty acids (PUFA) content was used as an oil phase, whereas Labrasol and sodium deoxycholate were used as surfactant and co-surfactant respectively. The *In situ* intestinal perfusion study exhibited sufficient increase in permeability and absorption characteristics by PTX-Cu-SNEDDs as compared to PTX-SNEDDs and PTX suspension. Therefore we have tried to highlight the most widely used class of agents (lipid vehicles and surfactants) in lipid based drug carriers which can tackle the synergistic effect of CYP3A4 and P-gp as shown as in Figure 2.^56^

## Lipid based carriers for anti-cancer delivery

**Figure 3:**
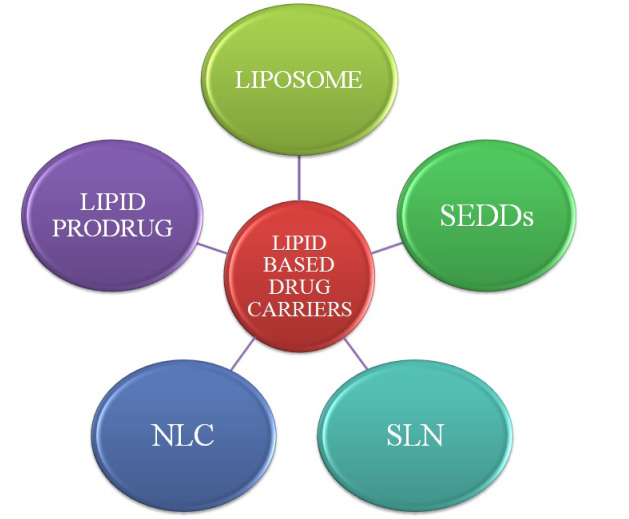
Schematic diagram of lipid based drug carriers.

Lipid based formulations have attained popularity as targeted, safe and effective delivery strategy for anti-cancer agents by overcoming the drawbacks of conventional drug delivery system during last five decades. Lipid based carriers system employed for anti-cancer drug delivery has been represented underneath in Figure 3.

## Liposome

**Figure 4:**
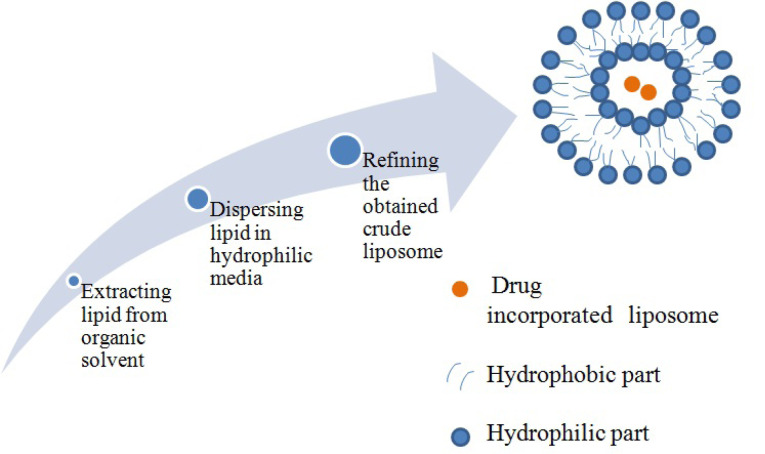
Generalized method for liposome preparation.

Liposome vesicle is bilayered, spherical in shape and usually composed of monomers with polar head and a non polar tail. The lipophilic head groups are oriented inside the bilayer. Liposomes were the first structured lipid based nanoparticle and possess unique characteristics to encapsulate both hydrophobic (at nonpolar lipid bilayer) and hydrophilic drug (at polar core). Their peculiar quality to permeate through the enterocytes and enhanced stability for drugs has gained sufficient interest for effective lymphatic drug targeting.^57^ Liposome preparation involves some generalized sequence of events as depicted in Figure 4.^58^

Ling et al^59^ reported that cefotaxime (having poor bioavailability and high water solubility) in liposomal formulation have enhanced bioavailability from 2.3 to 2.7 times compared to drug solution and physical mixture respectively, moreover the concentration of liposomal formulation was also substantially more in lymph thereby suggesting possible utility of liposome in targeting lymphatic vessels and addressing bioavailability related issues. Recent studies suggested that merely encapsulating the drug into liposomes may sometimes cannot provide sufficient lymphatic targeting, owing to other properties like the particle size, which governs the passage through bio-membranes.^60-62^ In an another study reported by Hashida et al^63^ liposomal formulation of carboxyfluorescein were inefficient in significantly permeating the mucosa of the intestine, but could enter intestinal mucosa when it was co-administered with lipid-surfactant mixed micelles as the latter could interact with intestinal luminal cell membrane, advocating the role of lipid based formulation with concomitant use of surfactants. Ye et al^64^ explored the potential of synthetic borneol as permeation enhancer in lymphatic targeting of 7-ethyl-10-hydroxycamptothecin incorporated nanoliposome (SN-38-Lips) and increase in lymph node uptake when administered through subcutaneous route. It was evident from their results that nanoliposomes when administered with 2mg/ml of Borneol exhibited enhanced lymphatic node retention and uptake.

## Liposome with coating material for enhanced performance

Liposomes with surface modifications have also been developed for combating issues of drug resistance, enzymatic metabolism and membrane permeability. Li et al^65^ have developed the formulation of polyethylene glycol (PEG)-coated liposomes of recombinant human epidermal growth factor. The area under the curve (AUC) enhanced form 1.7 to 2.5 folds which might have been an outcome of enhanced resistance towards enzymatic degradation and altered permeability.^66^ Iwanaga et al^67^ In an another study while examining the effect of coating on peptide drug (insulin as model drug) reported that coating the liposomal surface with polyethylene glycol, sugar portion of mucin increases the gastro-intestinal stability. Further, the coated liposomes were able to manage longer hypoglycemic effect over the uncoated liposomes. Moribe et al^68^ were able to increase the encapsulation of nystatin into liposome with distearoyl-N-(monomethoxy poly (ethylene glycol) succinyl) phosphatidylethanolamine (DSPE-PEG). The usefulness of liposome based products as delivery vehicle for anti-cancer drugs has been reflected by recent research reports as summarized in Table 1.

**Table 1:** Anti-cancer drugs benefitted by liposome

**Drug**	**Lipid Component**	**Interpretation**	**Special feature**	**Ref.**
Raloxifene hydrochloride	DSPC: Cholesterol, diethylene triamine penta acetic acid	Raloxifene loaded liposomal formulation provided effective uterine targeting as compared to non-liposomal drug.	Gamma scintigraphy studies depicted selective uptake of radiolabeled RLH.	69
Raloxifene	Dipalmitoyl phosph-atidylcholine, dioctyl phosphatidyl choline and calcium chloride. Dimethyl-β-cyclodextrin and sodium taurocholate	Raloxifene dimethyl-β-CD cochleate formulations were found to be successful in reducing breast tumors. Further matrix metalloproteinase-2 (MMP-2) enzyme was also found to be inhibited.	MCF-7 cell lines were used to evaluate antitumor activity.	70
Adriamycin	EPC/Chol 55:45 Chloroform, isopropanol, Water.	Adriamycin loaded liposome on local administration effectively inhibiting the proliferating cells by inducing apoptosis thereby reducing lymph node metastasis.	Highest apoptotic index of 21.73%.	71
Doxorubicin	Phosphatidylcholine, and cholesterol	Doxorubicin-loaded liposomes offered enhanced permeability and retention effect.	Passive targeting at cancer cells.	72

## Solid lipid nanoparticle (SLN)

**Figure 5:**
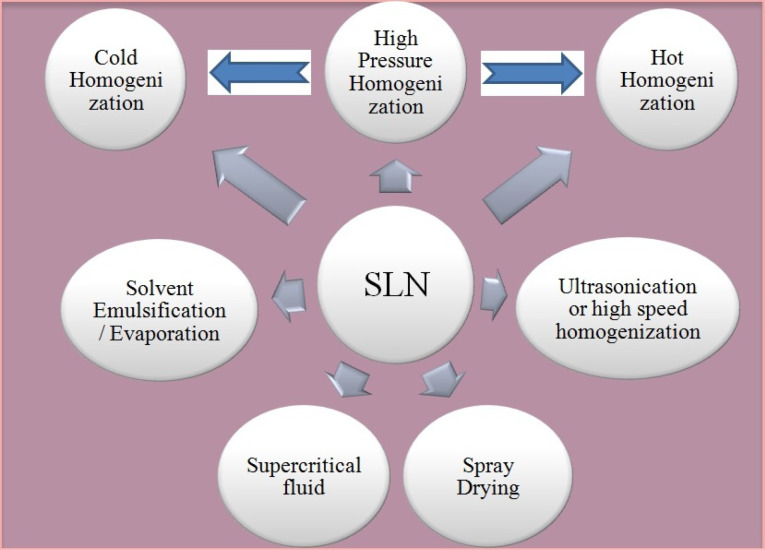
SLN preparation technologies.

SLN are lipid based drug carries which utilizes certain physiological lipids like mixtures of mono-, di-, or triglycerides along with surfactants (poloxamer and tweens). Moreover SLN are solid at room and physiological temperature. These unique characteristics offers reduced systemic toxicity and enhanced physical stability. Effective drug targeting (particularly to lymphatics) and increased drug encapsulation are additional benefits of SLN.^73-75^ The exclusion of biotoxic solvents during the manufacturing of SLN by high pressure homogenization (HPH) supports exclusive reduced biological toxicity.^76,77^ Most commonly explored technologies for SLN preparation has been depicted in Figure 5 and comparison in Table 2.

**Table 2:** Comparison of technologies employed for SLN/NLC preparation^78-82^

**Lipid Nanoparticle Technology**	**Methodology**	**Advantage**	**Disadvantage**
High pressure Homogenization	Hot Homogenization Drug incorporated lipid melt dispersed in surfactant rich hot aqueous phase, followed by homogenization under high pressure.	• Bulk manufacturing • High stability and loading of drugs.	• Not recommended for thermolabile drugs. • Drug may enter in aqueous medium under homogenization
	Cold Homogenization Drug dissolved in melted lipid, swiftly cooled under liquid nitrogen/dry ice, followed by milling. Then milled powder disperse in surfactant rich aqueous solution, followed by homogenization under high pressure.	• No drug partition in aqueous phase. • Thermolabile drugs can also be incorporated	• Surfactant involved may cause irritation/sensitization.
Solvent emulsification/evaporation	Lipid dissolved in organic solvent immiscible with water, followed by formation of an emulsion stabilized by surfactant. Solvent removal by solvent removal under reduced pressure.	• No thermal stress.	• Organic solvent used for dissolving lipid may possess toxicity. • Varied particle size of nanoparticles.
Super critical fluid technology	Supercritical fluids like carbon dioxide have been used for solvent extraction through o/w emulsions.	• Carbon-dioxide can serve as an alternative to other toxic organic solvents.	• Majority of organic solvents utilized are hazardous. • Large quantity of surfactant concentration required.
Ultrasonification	Lipid phase is dispersed surfactant rich aqueous phase, followed by homogenization under high shear/ultrasonication	• Simple manufacturing process.	• Polydispersed nanoparticles. • High concentration of surfactant.
Spray drying	SLN can be prepared from aqueous dispersion.	• Cost effective and substitute to lyophilization.	• Particles aggregation. • Lipids with melting point above 70° C can only be used

In comparison with other techniques, HPH can efficiently be explored for large-scale manufacturing of SLN. Homogenization can be done under hot and cold conditions. Both the process essentially involves dispersion/dissolution of drug into the lipid melt before HPH. Thereafter fluid is allowed to pass under high pressure (upto 200 bars) from a narrow passage in homogenizer. Dolatabadi et al^83^ employed simple homogenization technique to develop Alendronate SLN for inhalational use. The developed SLN formulation had particle size below 100 nm and low polydispersibility index 0.25. Furthermore the developed formulation exhibited better systemic bioavailability.Bakhtiary et al^84^ also employed hot homogenization technique to prepare Erlotinib SLN as dry powder inhalation product. Erlotinib SLN exhibited particle with spherical shape and size below 100 nm. As a model for non-small cell lung cancer human alveolar adenocarcinoma epithelial A549 cell lines were used for cytotoxic investigation, and the developed formulation exhibited higher anticancer activity.

## Role of SLNs in increasing anti-cancer drug absorption and permeability and cancer treatment

Eskandani et al^85^ successfully incorporated Shikonin-Act into SLN through hot homogenization method for enhancement in anti-proliferative activity of Shikonin-Act. Shikonin-Act loaded SLN exhibited better therapeutic potential against intact Shikonin in MTT (cytotoxicity) and comet assay (genotoxicity).In another study form Eskandani et al^86^ reported significant improvement in antiproliferative action of galbanic acid (GBA) by incorporating GBA into SLN. Hot homogenization method was used for preparation of SLN; the entrapment efficiency of developed SLN was more than 98%. GBA-SLN inhibited growth rate of A549 cells, moreover the cytotoxic effect of GBA-SLN was more prominent after 48 hours. Therefore result of their study depicted sustained and enhanced antiproliferative action form GBA-SLN.Findings from Hamishehkar et al^87^ were also in the agreement that sclareol (poorly water soluble) incorporated SLN was able to exhibit sustained (after 48 hours) and improved antiproliferative activity in A549 cell line.

Reports from the study conducted by Reddy et al^88^ on formulation of radiolabelled (^99m^Tc) etoposide loaded tripalmitin (ETPL) solid lipid nanoparticle depicted that among the three route explored for administration; subcutaneous, intraperitoneal and intravenous the tumor uptake specificity was highest after 24 hours in subcutaneous followed by intraperitoneal and intravenous administration which was 8-folds and 59-folds respectively, their finding suggested the specificity of ETPL in targeting lymphatic route associated metastasis.

Few recent research findings depicting the potential of SLN in enhancing anti-cancer drug efficacy are given in Table 3.

**Table 3:** List of anti-cancer drugs formulated as SLNs

**Drug Name**	**Lipid Components**	**Interpretation**	**Special Feature**	**Ref.**
Docetaxel	Trimyristin	Strong antitumor activity was observed from DCX-SLN, as compared to solubilized mixture of DCX in Tween 80/ethanol in cell culture and pre-established tumor in mouse model.	Decreased accumulation in vital organs depicted safety of the formulation.	89
Paclitaxel	Compritol 888 ATO, Precirol ATO5	Developed SLN of PTX could significantly enhanced in-vitro cancer cell death than Taxol, against MXT-B2 murine breast cancer cell line.	Drug entrapment efficiency was above 97%	90
Paclitaxel	Stearic acid, lecithin and poloxamer 188	SLN entrapped PTX showed enhanced cytotoxicity against PTX alone in cultured hepatocellular carcinoma cells.	PTX formulation exhibited sustains release with Higuchi kinetics	91
Paclitaxel	Folate-(PEG)-phosphatidyl-ethanolamine	Paclitaxel-loaded solid-liquid lipid nanoparticle showed enhanced rate of tumor inhibition on S180 tumor-bearing mice compared to PTX alone.	Sustained *in-vitro* release property	92
Paclitaxel	Glyceryl palmitostearate nanoparticle	Anti-proliferative activity was intact for PTX against B16F10 cell lines in chemosensitive assay.	Mean particle size of the formulation was (172-253 nm)	93
Paclitaxel	PEG and folate modified monostearin SLN	Cellular uptake and cytotoxicity of PTX was enhanced through folate and PEG modified SLN.	Octadecylamine–fluorescein isothiocyanate was used to formulate fluorescent SLN.	94
Methotrexate	Sodium taurodeoxy-cholate, soya lecithin and stearic acid	The survival time of Ehrlich ascites carcinoma suffering mice was increased owing to better anti-cancer activity of methotrexate-SLN.	MTX-SLN was reported to have average particle size of 270 nm with 51.3% of drug loading.	95
Vinorelbine bitartrate	PEG2000-stearic acid	PEG modification of SLN inhibited phagocytosis of VB-pSLNs by RAW264.7 cells as well as increased cellular uptake by MCF-7 and A549 cell lines.	Octadecylamine–fluorescein isothiocyanate was used as florescent marker in cellular uptake studies.	96
Camptothecin	Poloxamer 188, stearic acid, soya lecithin	The mean residence time and area AUC of camptothecin loaded SLN was higher as compared to camptothecin solution.	Camptothecin-SLN has 196.8 mean particle size with -69.3 mV of zeta potential.	97
Tamoxifen citrate	Glycerol behenate, sodium tauroglyco-cholate	Tamoxifen citrate incorporated solid lipid nanoparticle exhibited 3 fold and 3.5 fold increase in t (1/2) and mean residence time respectively.	Smaller mean diameter particle was obtained with homogenization at 15 000 psi for 3 cycles of SLN.	98
5-Flurouracil	Dynasan, soyalecithin, polyvinyl alcohol*	5-flurouracil (5-FU) incorporated SLN could sustain delivery up to 48 hours.	Mean particle size of 402.5 nm ± 34.5 with PDI of 0.005	99

* As stabilizer

## Nano-structured lipid carriers (NLCs)

The SLN suffered from multitude of drawbacks particularly of insufficient drug loading capacity and leaching of drug during storage (alteration to β type); thereby lipid-based nano-carriers were fabricated to address the shortcomings of SLN, using both solid and liquid characteristics of lipids.^100^ This peculiar characteristic offers better drug entrapment during product shelf life, which in turn increases drug solubilizing potential of NLCs system. Studies have also been conducted for exploring the potential of SLN for targeted and sustained delivery of drugs. Moreover judiciously formulated NLCs system (tailoring the composition and formulation processing parameters) can be used for effective intestinal absorption of drugs which can synergistically improves drug bioavailability.^101^ Therefore NLCs are categorized as second generation lipid based nano-drug carriers.^102-106^ Dolatabadi et al^107^ successfully prepared Ketotifen incorporated NLCs with enhanced systemic exposure and reduced cellular toxicity. Hot homogenization and ultrasonication method was used for NLCs preparation. Ketotifen loaded NLC has particle size below 100 nm with high encapsulation efficiency of 70%.

## NLCs and their role in increasing anti cancer drug absorption and permeability

Delivery of drug substances with poor bioavailability owing to their low aqueous solubility, permeability and high metabolic activity has been a herculious task for the formulation scientist since long. Majority of problems can be addressed by incorporating them into NLCs such as fenofibrate, isoliquiritigenin, baicalin.^108-110^ Metabolism of CYP3A4 substrate drug can be prevented by using surfactant (polysorbates, cremophors, and poloxamer) which are inhibitor of the CYP3A4 activity. Moreover conversion of drug physical state from crystalline to amorphous enhances dissolution velocity and increases the uptake of drug by lymphatic system. Making an overall enhancement in drug absorption and bioavailability.^111-114^ The entrapment of drug into the lipid matrix provides stealth effect to photolabile and also to drug prone to hydrolytic degradation. Tailoring the globular size of NLCs in the range from 120 to 200 nm reduces the uptake by RES, thereby providing effective enhancement in oral bioavailability.^115,116^ Ding et al^117^ reported the usefulness of NLCs in co-delivery of PTX and indocyanine green (ICG). The developed formulation have dual characteristic of chemo as well as photodynamic therapy. ICG was reported to enhance the drug release by laser irritation, further increased intracellular uptake and cytotoxicity was obtained by concomitant use of PTX and ICG.

## Limited lymphatic uptake of NLCs

Drug incorporated NLCs which are tailor made convert into mixed micelles on interaction with bile salts in the GIT, making them capable of gaining access into lymphatic system thereby circumventing the metabolic activity of liver. The limited lymphatic access also serves the purpose of dose frequency reduction, enhanced concentration of anti-cancer drug at localized and metastatic tumor cells. These factors advocate effective management in cancer chemotherapy and associated side effects.^118^ Anti-cancer drugs benefitted by NLCs are shown in Table 4.

**Table 4:** List of anti-cancer drugs formulated as NLCs

**Drug**	**Lipid vehicle**	**Interpretation and remark**	**Ref.**
Docetaxel	Soy lecithin, glyceryl monostearate, and fatty acids	Prepared DTX-NLC (murine melanoma treatment) accounts for lower toxicity in therapeutics dose and selective cytotoxicity to A549 cells through apoptosis.	119
Docetaxel oleate	Poloxamer F68, oleic acid, and Glyceryl monostearate	Prepared prodrug NLC of Docetaxel exhibited improved membrane permeability in in-situ single pass intestinal perfusion and 4.04 fold increase in bioavailability	120
Biochanin A	Glycerol monostearate, medium chain triglyceride, soya lecithin, and Tween 80*	Biochanin loaded NLC exhibited higher AUC values with prolonged in-vitro residence time	121
Tamoxifen citrate	Poloxamer 188, glyceryl monostearate, Labrafil WL 2609 BL, Tween 20*, and Tween 80*	Similar cytotoxic activity was observed both on human (MCF-7) and mice (4T1) breast cancer cell lines form Tamoxifen and Tamoxifen-NLC	122
Tamoxifen citrate	Glyceryl monostearate, Labrafil WL 2609 BL,Tween20*, Tween 80*, Poloxamer 188*	Tamoxifen loaded NLC exhibited 2.71 and 7.10 fold increment in bioavailability and T_1/2_ respectively, also measurable amount was detected in mesenteric lymph node.	123
Etoposide	Glyceryl monostearate, soybean oil, soya lecithin	Etoposide loaded-DSPE-NLC was highly cytotoxic to human epithelial-like lung cell carcinoma cells, and even relative bioavailability was 3.5 fold to Etoposide-suspension	124

*Emulsifier.

## Self-Emulsifying Drug Delivery Systems (SEDDs)

SEDDs are complex mixture of Lipid, Surfactant and Co-surfactant the later may or may not be used. Lipid Based drug delivery system have emerged from simplistic oily solutions; they can be either self-micro emulsifying drug delivery system (SMEDDs) or self-nanoemulsifying drug delivery systems (SNEDDs). Whether SEDDs, SMEDDs or SNEDDs they have unique characteristic to self-emulsify into oil-in-water (O/W) emulsion *in-vivo* on subsequent dilution and agitation by the physiological system.^125-128^ SEDDs formulations have proven their utility in addressing the issue of bioavailability for BCS Class II and IV candidates and essentially comprises of lipid globule size in the range of 200 nm-5 mm.^129-131^ When the globular size of the inner phase ranges from 100-200 nm they are termed as SMEDDs and makes transparent micro-emulsions on dilution. Whereas, those self emulsifying lipid based formulations having lipid globular size below 100 nm are categorized as SNEDDs.^132,133^

A plethora of research article have been published which clearly exhibits the usefulness of SMEDDs and SNEDDs for lymphatic route targeting. As the drug is in pre-solubilized state into the lipid component it presents drug in ready to be absorbed condition, it also circumvents the effect of metabolic enzymes and drug effluxer, therefore contributes to overall enhancement in drug bioavailability.^134,135^ The faith of drug substance incorporated into lipid based drug delivery system *in-vivo* is dependent on the factors like physiochemical properties of drug, nature of lipid used (SCT, MCT or LCT). Therefore, judicious selection of surfactant and co-surfactant blend and its ratio is required as it is a guiding process in drug absorption into the luminal lymphatic pathway and effective drug concentration to tumor cells.^136-138^ The type of oils used for the formulation of SMEDDs is given in Table 5. Anti-cancer drugs benefitted by SEDDs and SMEDDs are given in Table 6.

**Table 5:** List of lipids explored in formulation of SMEDDs^34^

**Type of Lipid**	**Description**
Long chain triglycerides	Sunflower oil, Sesame oil, Soybean oil, arachis oil, Rice bran oil, castor oil, cottonseed oil, maize (corn) oil, canola oil, hydrolyzed corn oil, triolein olive oil, safflower oil, palm oil, peanut oil,
Medium chain triglycerides and related esters	Crodamol GTCC®), Caprylic/capric triglycerides (Akomed E, Akomed R, Miglyol 810, and Captex 355, Captex 200, Triacetin Neobee M5®, fractionated coconut oil (Miglyol 810 and 812 ), Labrafac CC, Captex 300,
Medium chain mono and di-glycerides Long-chain mono glycerides Propylene glycol (PG) fatty acid esters	Mono and diglycerides of capric/caprylic acid. (Capmul MCM and Imwitor), Glycerylmonooleate (Capmul GMO, Peceol), glycerylmonolinoleate (Maisine-CC/Maisine 35-1) PG Diester of caprylic/capric acid (Labrafac PG), PG monocaprylic ester (Sefsol-218), PG monolaurate (Lauroglycol FCC, Lauroglycol90, Capmul PG-12), PG dicaprylate (Miglyol 840)

**Table 6:** List of anti-cancer agents benefited by SEDDs/SMEDDs

**Drug**	**Oil**	**Emulsifier/Co-Emulsifier**	**Outcome**	**Ref.**
Docetaxel	Glyceryl tricaprylate	Cremophor RH 40, diethylene glycol monoethyl ether; HPMC for promoting super saturation	Docetaxel loaded SEDDs formulation exhibited 8.77 folds higher AUC	139
Doxorubicin and LYP-1 (anticancer peptide)	Maisine/Peceol	PEG 300, TPGS, Gelucire 44/14, Labrasol, Tween 80 propylene glycol	Co-administration of doxorubicin with LYP-1 through SMEDDs formulation showed reduction in tumor growth and metastasis	140
Etoposide	PC-complexed; octyl and decyl mono-glyceride	Cremophor EL and PEG 400	60-fold increase in bioavailability	141
9-Nitrocamptothecin (9-NC)	Ethyl oleate	Tween-80/ Cremophor EL and PEG-400/ethanol	SMEDDs based formulation produced better tumor reduction as compared to oral suspension	142
Exemestane	Capryol 90	Cremophor ELP and Transcutol P and	Exemestane loaded formulation exhibited 2.8-fold higher oral bioavailability against un emulsified formulation.	143
Raloxifene	Mono and di glyceride of capric/ caprylic acid Capmul MCM C8	Akrysol K-140, PEG-200	In-vitro intestinal permeability studies exhibited enhanced permeability as compared to drug suspension.	144
Mitotane	Capryol 90	Tween 80 and Cremophor EL	The optimized mitotane formulation exhibited better permeability from intestinal barrier against mitotane solution (14.85 ± 0.8 vs. 3.03 ± 0.2 μM/cm 2 ), together with 3.4-fold higher bioavailability	145
Etoposide	Medium chain triglyceride	Polyoxyethylene sorbitan monooleate-20, diethylene glycol monoethyl ether, propylene glycol monolaurate type-I	The % drug release was 1.6 and 1.4 folds for optimized formulation in simulated gastric fluid and simulated intestinal fluid	146
Curcumin	Ethyl oleate,	OP: Cremophor, (PEG 400), isopropyl myristate, Cremophor RH40R,	The Curcumin loaded SMEDDS absorption was 3.86 times or 12.73 times compared with a Curcumin suspension.	147
Curcumin	Castor oil	Tween 80 and ethanol	Curcumin solubility in SEDDs was 1.93 mg/mL	148
Curcumin	Ethyl oleate	Transcutol P Cremophor RH 40	SMEDDs based formulation was targeted for colonic delivery through pulsatile capsule.	149
Curcumin	Lauroglycol	Labrasol and Transcutol HP	The C_max_ and AUC for solid SEDDs was 4.6 and 7.6- folds higher	150
Curcumin (Type IV lipid based formulation)	Gelucire 44/14	Labrasol, Vitamin E TPGS and PEG 400	Lipid based oral formulation exhibited enhanced C_max_ and AUC0-35.8-fold increase in the oral bioavailability as compared to control	151
Curcumin	Isopropyl myristate	Cremophor RH 40 and ethanol	Curcumin SMEDDs relative bioavailability was 1213% as compared to Curcumin oral suspension	152
Paclitaxel	Vitamin E	DOC-Na, TPGS, Propylene glycol, Cremophor RH 40	On use of P-gp inhibitor (Cyclosporine-A) oral absorption of Paclitaxel was enhanced	153
Paclitaxel	Glyceryl dioleate	Cremophor EL, PEG 400; HPMC as super saturation Promoter	5-fold higher bioavailability	154
Paclitaxel	DL-alpha tocopherol	TPGS, tyloxapol, DOC-Na	5 fold increase in Paclitaxel loading as compared to i.v. formulation with less toxicity.	155

## SNEDDs for hydrophobic drugs

As the absorption of drug substance (hydrophobic) is the limiting factor owing to crystalline structure of the molecule, the entire dose of drug in SNEDDs exists in dissolved state in the lipid concentrate, therefore overall enhancement in absorption of the drug substance takes place. This finally promotes partitioning through the enterocytes and into the systemic circulation.^156-160^

Furthermore the lipid based formulations (emulsified in the stomach) or lipid digestion products after reaching to the enteric system (duodenum) instigate bile juice secretion (bile salts and biliary lipids) stored in the gall bladder. The composition of bile juice is such that it supports further processing of lipid based formulations as they act as a medium for the solubilization of hydrophobic fatty acids, mono and di-glyceride which are incorporated in series of colloidal structure at intestinal lumen. The hydrophilic part of the micelles orients into the aqueous phase whereas hydrophobic chain forms the central part.^161-163^

The overall solubilizing potential of the intestinal lumen is enhanced by the formation of mixed micellar phase, through lipid based formulations reducing precipitation of hydrophobic drugs. Their solubilizing potential is related with the ability to utilize enormous interfacial surface where the hydrophobic drug partitions.^164,165^

## SNEDDs as remedy for CYP3A4, P-gp substrate drugs

The mechanistic approach behind enhanced oral bioavailability of SNEDDs based formulation as hypothesized by many researchers might be an outcome of enhanced permeability by trans-cellular route, as the formulation components associates with trans-cellular membrane makes it more fluidic which in turn provide massive passive permeation of formulation components.^166,167^ The other theories have originated from the functionality of lipid based excipients; as they have ability to inhibit the efflux transporters making conformational changes to the efflux causing pumps (p-gp) thereby enhancing drug concentration at the target site.^168,169^ Conventional theory advocated the dominant role of liver in pre-systemic metabolism of drugs whereas the newer finding suggests that intestinal lumen is a major obstacle for drug substance permeability. This hypothesis was generalized on experimental findings which reflected high level of concentration of proteins associated with drug metabolizing enzymes in liver rather than small intestine.^170^ Though CYP3A associated drug metabolizing enzymes were largely expressed at the developed villus tip of enterocytes in the small intestine.^171^ The CYP3A sub-family of enzymes is considered to have a major role in Phase I metabolism in human beings. In fact, the CYP3A sub-family is responsible for the oxidative metabolism of ~50% of currently marketed drugs.^172^ Recent research done by several researchers has shown that more than 70% of the enzymes associated with CYP3A4 prevails in small intestine, whereas its prevalence in hepatic was comparatively low to 30%, which somewhat have demystified the dominant role of liver in first pass effect.^173^

The SNEDDs formulation serves the purpose of delivering hydrophobic drugs by presenting the drug in pre-solubilized state in nano sized globules which are thermodynamically stable with enhanced absorptive potential.The overall bioavailability is also enhanced owing to reduced pre-systemic metabolism by CYP3A4 and reduced or negligible P-gp mediated drug efflux.^174^ The role of CYP3A4 and P-gp was discussed when addressing low bioavailability of cyclosporine by Wu et al^175^ a BCS Class II compound and P-gp, CYP3A4 substrate. The SNEDDs formulation exhibited better permeability through membranes when administered in solubilized state with corn oil as lipid vehicle, but reduced bioavailability was observed owing to higher metabolism by CYP3A4 and also as it was highly effluxed out from the cells. These suggestions were in agreement that merely solubilizing the drug in lipid do not suffices the condition of poor bioavailability as the incorporation of vehicles that can inhibit the effect of P-gp and CYP3A4 is also equally important. Akhtar et al^176^ developed SNEDDs of etoposide (a p-gp substrate), and reported higher permeability coefficient in apical to basolateral direction from optimized SNEDDs formulation across Caco-2 monolayers as 2.6 and 11-fold in comparison to marketed formulation and lone drug solution respectively. Enhanced permeability and bioavailability has been attributed to Cremophor RH 40 (as surfactant) ability to inhibit the P-gp efflux activity in the gut and ability of Transcutol P (as co-surfactant) to alter membrane permeability. The SNEDDs has been explored for maximizing the therapeutic potential of anti-cancer drugs and results of their study are listed in Table 7.

**Table 7:** List of anti-cancer agents benefitted by SNEDDs

**Drug**	**Lipid Components**	**Interpretation**	**Ref.**
Etoposide	Oleoylmacrogol-6-glycerides, Gelucire 44/14, Peceol, Transcutol** P, Plurol Oleique and Labrasol*	The formulation showed better transportation of etoposide across the Caco-2 cell lines and higher cytotoxicity than the Etosid® and free drug against A549 human epithelial lung carcinoma cell lines	176
Docetaxel	Capryol 90, Labrasol*, and Transcutol HP**	17% higher oral bioavailability for D-SNEDDs as compared to 2.6 % for Docetaxel solution, with high anti-tumor efficacy and reduced toxicity.	177
Tamoxifen citrate	Maisine 35-1, Capryol 90, Cremophor RH40*, propylene glycol**	Higher drug release from SNEDDs as compared to Tamoxifen citrate suspension.	178
Docetaxel	Maisine 35-1and Capmul MCM with Tween 80* and Transcutol-HP**	Self Nano Emulsifying Lipidic Nano-micelles System (SNELS) Formulation enriched with PUFA (Long Chain Glyceride) exhibited better dissolution, enhanced p-gp inhibition and reduced pre-systemic metabolism as depicted from *in vivo* pharmacokinetic studies, better permeability and absorption was also reflected from *ex-vivo* permeation and*in situ* perfusion studies. Moreover LCG based formulation were highly targeted to lymphatic system as compared to MCG (medium-chain fatty acid glyceride)	179

*Emulsifier, **Co-emulsifier.

## Lipid prodrug

**Figure 6:**
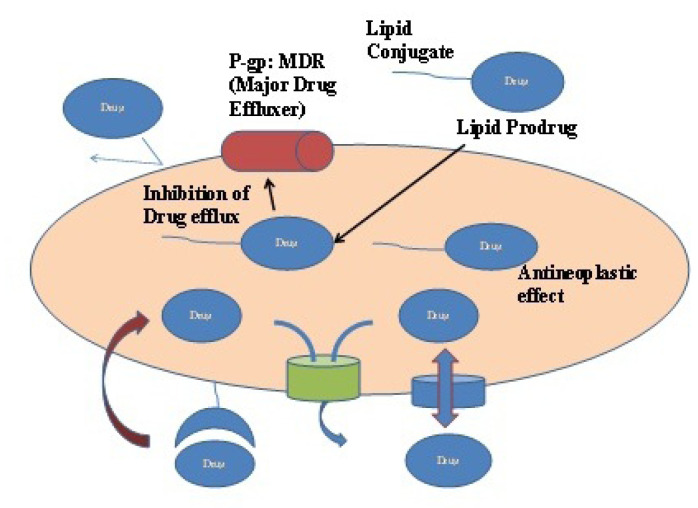
Lipid prodrug can effectively reduce drug efflux from P-gp and MDR.

A plethora of lipid carriers have been investigated for the development of lipid prodrug which are generally a unique mixture of glyceride, fatty acids, and phospholipids. The drug is linked either at the carboxylate group or at the ω-position of the carbon chain at its terminal part.^180^ A Glyceride conjugation is an outcome of attaching carboxylate group of drug moiety through ester linkage, whereas the drug can also be linked through phosphate group or at the glycerol backbone. The enhanced permeability exhibited by drugs attached to the glycerol backbone is attributed to their ability to permeate apical membrane of the intestinal lumen and the blood brain through natural absorption pathway of phospholipids.^181^ Drug-lipid conjugations can reduce total quantity of drug being effluxes out from the cells by P-gp or MDR transporters which in turn will increase permeation to cancerous cells and prolonging the retention of anti-cancer agents Figure 6.^182^ Numerous lipid-taxane conjugates, with alterations at 2 or 7-OH position of series of second generation taxoids (Paclitaxel, Docetaxel SB-T-1103, SB-T-1104, SB-T-1213, SB-T-1214, SB-T-1216, and SB-T-1217) have also been investigated.^183^

## Targeted lipid nano-carriers for cancer therapeutics

Targeting anti-cancer drugs to proliferating cells through lipid based delivery systems have proven benefits in selectively targeting the cancerous cells, avoidance of exposure to healthy cells, hydrophobic drug delivery and prolonged exposure over conventional chemotherapy strategies which have dose dependent side effects due to their non-selective bio-distribution.^184^ Targeted delivery of anti-cancer drugs can be achieved either by active or passive targeting. The active targeting is achieved by attaching lipid based nano-carriers with tumor specific binding ligand. These specific bindings results in selective accumulation at target site. Whereas, passive targeting approach delivers the drug cargo passively to proliferating cells utilizing the rapid vascularization of hyper-permeable cells. The nano-particulate size (10-100 nm) and its surface properties (hydrophilic) are decisive in passive targeting and assembly within tumors.^185-187^ Jain et al^188^ successfully investigated tumor targeting specificity of Ferritin integrated 5-flurouracil SLN. Fr-SLNs showed 7.7-folds more binding affinity with MDA-MB-468 breast cancer cell lines in contrast to non Ferritin integrated SLN of 5-FU under *in-vitro* studies. Further under therapeutic investigation in MDA-MB-468 cancer induced mice, formulation with Fr-SLNs exhibited significant reduction in tumor growth as compared with plain 5-FU and non-Ferritin integrated SLN of 5-FU.Yang et al^189^ explored target specificity to CD44 overexpressing tumors with PTX incorporated SLN having hyaluronic acid-coating. Melt extrusion technique was employed for preparing cationic PTX-NLC, which were further coated with hyaluronic acid. Tumor specificity, pharmacokinetics and biodistribution were investigated in B16-bearing Kunming mice. It was concluded from their research that Hyaluronic acid coated SLN of PTX exhibited tumor targeting efficiency of 14.46 %, which was around 1.4 fold higher against Taxol® and drug circulation was also enhanced from 3h to 6h. Liu et al^190^ successfully explored synergistic anticancer action against cervical cancer from engineered SLN with trans-activating transcriptional activator (TAT) for co-delivery of PTX coupled with α-tocopherol succinate-cisplatin prodrug (TOS-CDDP) (TAT PTX/TOS-CDDP SLNs). The *in-vitro* and *in-vivo* studies were in agreement that developed formulation showed specific antitumor action on HeLa cells and cervical cancer suppression with reduced toxicity in vivo. Furthermore to get an insight into the characteristics of different nano-particles (polymeric, metallic) in comparison with lipid based nano-carriers for cancer therapeutics we have summarized recent research trends in the form of a comprehensive Table 8.

**Table 8:** Comprehensive table showing different characteristics of nano-particles with special emphasis on lipid based drug carriers

**Nano-carrier types**	**Drug**	**Composition**	**Cancer Type/ Cell lines investigated**	**Formulation Characteristics**	**Outcome**	**Ref.**
SNEDDs	Sunitinib malate (SM)	BLauroglycol-90 (oil), Triton- X100 (surfactant) and Transcutol-P (cosurfactant)	HT-29 colon cells	PS* 42.3 nm, PDI** (0.174), ZP*** value (-36.4mV)	Optimized SNEDDs formulation was about two times more efficacious in comparison to plain SM	191
SNEDDs	Etoposide	GT and PGMCE (1:1) Cremophor RH 40, Transcutol P	For cytotoxicity A549 human epithelial lung carcinoma cell lines, and Caco-2 cell monolayers for transportation	PS* 36.01 nm, ZP*** (-27.1mV)	AUC was increased to 7.9 fold compared to etoposide drug suspension	176
SLN	Galbanic acid	glyceryl palmitostearate (Precirol ATO 5), Poloxamer 407	A549 cells	PS* 92nm, ZP*** value( -23.39mV), Entrapment efficiency- >98 %	Sustained and enhanced antiproliferative action form GBA-SLN	86
SLN	Paclitaxel	E.wax and Brij 78	U-118 and HCT-15 cell lines, brain uptake in situ rat brain perfusion model	PS* <100 nm, with high drug loading.	Significant modulation of p-gp at Blood brain barrier by Brij 78	192
Targeted-NLC	Docetaxel	1,2-Distearoyl-sn-glycero- 3-phosphoethanolamine-N- [amino(polyethylene glycol)-2000]	Murine model bearing B16	PS*168nm, Encapsulation efficiency >95%	Increased accumulation of drug in both tumor and tumor vasculature, therefore successful targeting to VEGFR-2	193
SMEDDs	Curcumin	Semi-synthetic oleic acid derived bicephalous heterolipid, E1E, Solutol HS-15, Transcutol HP	Human cervix cancer cell line HeLa	PS* 22.39 nm, PDI** 0.243, Curcumin loading efficiency 70.52	26 fold increment in absorption	194
SMEDDs	Dutasteride	Capryol^TM^ 90, Cremophor® EL, Transcutol® HP	Pharmacokinetic studies in Male Sprague–Dawley rats	PS*35.3nm	10.5 and 13.2 fold increment in AUC _0→24h_ and C_max_ respectively	195
Amorphous polymeric NPs	Cisplatin	P(3HV-co-4HB)-b-mPEG amphiphilic block copolymer	MTT assay using DU145 cell line	PS* 155 nm, PDI** 0.154, ZP*** value (-18mV)	The polymeric nanoparticles of cisplatin exhibited sustained drug delivery	196
PLGA-based nanoparticles	Paclitaxel	PEGylated PLGA-based nanoparticles	MTT assay on Human Cervix Carcinoma cells (HeLa)	PS* 112 nm, PDI** 0.18, ZP*** (-0.556 mv)	The developed nanoparticle exhibited significant anti-proliferative activity compared to Taxol on HeLa cell lines.	197
Carbon nanotubes	Doxorubicin	Anti body P-gp functionalized hydrophilic single walled nanotubes	Multidrug resistant leukemia (K562R) cells	Effective loading and targeted delivery of doxorubicin	Significant anti-proliferative activity in K562R leukemia cells with 2.4 times higher cytotoxicity	198
Cyclodextrin based Nanoparticles	Docetaxel	Poly(e-caprolactone) (PCL) and poly(ethylene glycol)-block-poly(e-caprolactone) (mePEG-PCL) nano particles with hydroxypropyl-β-cyclodextrin (CD) coating	MCF-7 human breast adenocarcinoma cell lines	PS* 60-132 nm, ZP*** (-22 and -37mV) , Encapsulation efficiency ranges from 46 and 73%	hydroxypropyl-β-cyclodextrin (CD) and PCL based nanoparticles exhibited significant antiproliferative activity against MCF-7 human breast adenocarcinoma cell lines	199
Superparamagnetic iron oxide nanoparticles (SPIONs)	Doxorubicin	polyamidoamine (rPAA) with poly(ethylene glycol)(PEG)/dodecyl amine graft	Xenograft MDA-MB-231 breast tumor in mice	PS* _˜_150nm	rPAA@SPION effectively inhibited tumor growth in xenograft MDA-MB-231 breast tumor induced mice	200
Gold nanoparticle	Oxaliplatin	Functionalized with a thiolated poly(ethylene glycol) (PEG) monolayer capped with a carboxylate group	A549 lung epithelial cancer cell line and colon cancer cell lines HCT116,HCT15, HT29, and RKO	PS* 30-40 nm,	Functionalized NP exhibited 6 fold increments in cytotoxicity in A549 lung epithelial cancer cell line and 5.6-fold more cytotoxic in colon cancer.	201

*PS = Particle Size, **PDI = Polydispersibility index, ***ZP = Zeta Potential, VEGFR-2 = Vascular endothelial growth factor receptors, UGT = UDP-glucuronosyltransferase

## Conclusion and future prospects

Lipid based formulation have emerged as a panacea for anti-cancer drug therapeutics. Lipid component as delivery vehicle for hydrophobic drugs have been successfully utilized for enhancing their oral bioavailability. Moreover, lipid component like Peceol, Maisine 35-1 and Gelucire 44/14 have proved their usefulness in lymphatic system targeting and effective P-gp and CYP3A4 modulatory effect. Further, as per our discussion in this article the judicious selection of lipid components is advocated as selecting long chain triglyceride over short and medium chain triglycerides when lymphatic drug targeting is desired. Therefore, lipid based formulation can be employed for lymphatic voyage of anti-cancer drugs with additional benefits of P-gp and CYP3A4 modulation for drugs having poor hydrophilicity, low intestinal permeation and high CYP3A4 mediated metabolic activity.

## Ethical Issues

Not applicable

## Conflict of Interest

The authors declare no conflict of interest, financial or otherwise.

## Acknowledgments

The manuscript was not financed from any source.
